# The Influence of Friction on Design of the Type of Bracket and Its Relation to OHRQoL in Patients Who Use Multi-Bracket Appliances: A Randomized Clinical Trial

**DOI:** 10.3390/medicina57020171

**Published:** 2021-02-17

**Authors:** Adriana González-Sáez, Laura Antonio-Zancajo, Javier Montero, Alberto Albaladejo, María Melo, Daniele Garcovich, Alfonso Alvarado-Lorenzo

**Affiliations:** 1 Faculty of Medicine, University of Salamanca, Avenida Alfonso X el Sabio s/n, 37007 Salamanca, Spain; adrianagonzalezsaez@gmail.com (A.G.-S.); javimont@usal.es (J.M.); albertoalbaladejo@hotmail.com (A.A.); alfonsoalvaradolorenzo@gmail.com (A.A.-L.); 2 Conservative Dentistry and Endodontics, Department of Stomatology, University of Valencia, Gascó Oliag 1, 46010 Valencia, Spain; maria.melo.alminana@gmail.com; 3 Department of Dentistry, European University of Valencia, Passeig de lÁlbereda, 7, 46010 Valencia, Spain; daniele.garcovich@universidadeuropea.es

**Keywords:** orthodontics, pain, quality of life, low-friction brackets, oral health

## Abstract

*Background and objectives:* The aim of this study was to evaluate the influence of friction on design of the type of bracket, patients’ perception of pain and the impact on their oral health-related quality of life. *Materials and Methods:* A randomized clinical trial was carried out with 90 patients (62.2% women and 37.8% men) with three kinds of fixed multi-bracket appliances: Conventional (GC), fixed multi-bracket low friction (GS) and self-ligating (GA). The VAS (Visual Analogue Scale) was used to determine pain during the first seven days of treatment at different points in time. The patients were also given the OHIP-14 (Oral Health Impact Profile) questionnaire to analyse their oral health-related quality of life (OHRQoL) after the first 30 days of treatment. The ANOVA test was used for the analysis of the variables and the post hoc Bonferroni test for the comparison between groups. *Results:* Maximum pain was observed between one and two days after the start of treatment. The GC group showed the greatest degree of pain, with maximum values (4.5 ± 2.0) at 24 h. The self-ligation brackets show lower impact on patients’ oral health-related quality of life (0.8 ± 2.2, *p* < 0.01). *Conclusions:* Friction in the type of bracket influences pain and the Oral Health-Related Quality of Life of patients who use multi-bracket fixed orthodontics.

## 1. Introduction

Controlling friction in orthodontics is a challenge, because it can be a negative influence by impeding tooth movement [1]. Friction is associated with the sliding mechanics that occur in spaces closure in particular. Another relative form of sliding is that which occurs in the first phases of multi-bracket orthodontic treatment, alignment and levelling, with flexible and reduced section super-elastic round arches, which is called indirect sliding. Both forms of sliding create potential friction [2].

In the literature, we can find a multitude of variables that may directly or indirectly contribute to friction levels in the bracket–arch interface. The variables range from physical factors (properties of the wire, brackets, ligature type, bracket–arch, etc.) to biological factors (tooth misalignment, intraoral forces, stick-slip phenomena, saliva, acquired film, etc.) [1,3].

Insofar as brackets, differences in friction can be found that depend on the material the brackets are made of, their width, shape and slot geometry and the design of the bracket itself. It has traditionally been thought that part of the pain patients feel could be due to the friction and forces present during orthodontic treatment [4,5,6].

The majority of the authors studied believe that treatments with self-ligating (low-friction) brackets lead to patients feeling less pain during the initial phases of treatment [7,8]. However, other studies came to the conclusion that greater pain is perceived during the clinical manipulation process with patients when the arches are inserted and deinserted [9]. Some studies conclude that self-ligating patients (Damon 2 and 3^®^) would perceive a lower average intensity of pain [10,11], while other studies did not find evidence that the self-ligating brackets are associated with less discomfort than conventional brackets during the initial phases of treatment with a fixed appliance, independent of age or sex [12].

Synergy is a special conventional low-friction bracket that, because of its design, can be used with an elastic or metal ligature in a conventional way with six different ways of ligating [13].

According to conflicting data in the scientific literature, in this study we wanted to evaluate the influence of friction on design and the type of bracket and the pain perceived by the patient and its relationship with their oral health-related quality of life (OHRQoL) in the initial stage of orthodontic treatment. The null hypothesis was that there is no difference between conventional, conventional low-friction and self-ligating brackets insofar as regarding pain and OHRQoL in patients using fixed multi-bracket orthodontics.

## 2. Experimental Section

### 2.1. Study Design

A randomized study was done using a sample of 90 patients split into groups of 30 patients each. The first group (GC) used conventional vestibular brackets (Diamond plus^®^, Cimbis Orthodontics, Madrid, Spain). The second group (GA) used self-ligating vestibular brackets (Bio-smile^®^, Cimbis Orthodontics, Madrid, Spain). The third group (GS) used conventional low-friction brackets (Synergy^®^, Denver, CO, USA). The same type of bracket slot (0.022″) was used in all three groups.

The study was conducted according to the ethical standards set by the Declaration of Helsinki for Biomedical Research and the protocol was approved by the Bioethics Committee of the University of Salamanca (USAL_20/516). All the patients who participated in the study were informed about the procedures to be followed and were provided with informed consent for their approval.

### 2.2. Sample Size Calculation

Previously carried out studies were considered to calculate the sample size [7,8,10,11,12]. With a margin of error of 5% and confidence level of 95%, it was determined that the optimum size of the object sample was 110 patients, including 10% abandonment. In this case, the study sample was 110 patients but 20 were excluded, so we ultimately split the 90 patients into three groups of 30 people each.

### 2.3. Patient Selection

**Figure 1:**
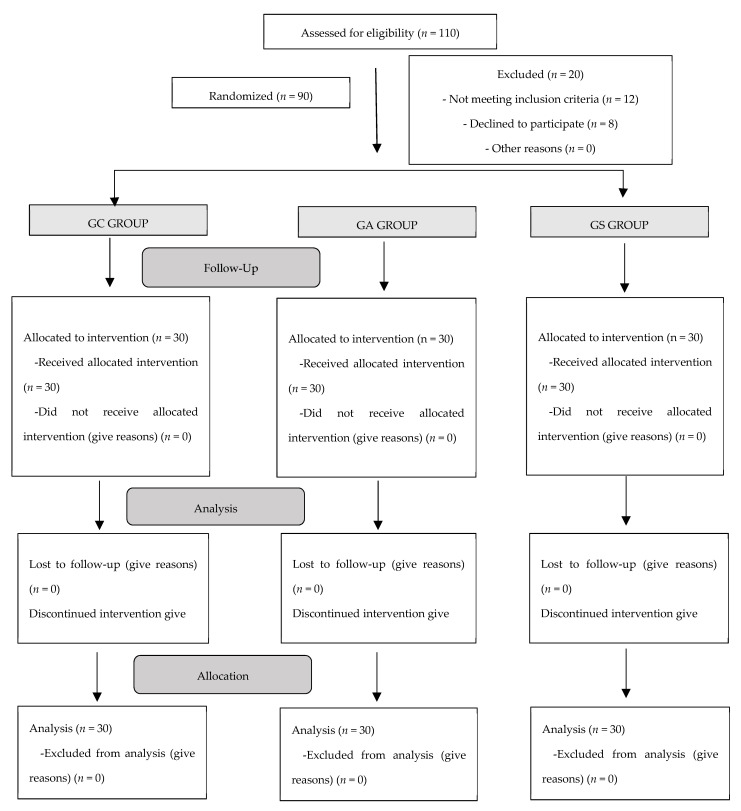
Flowchart for randomized clinical trials (CONSORT, 2010: Consolidated Standards of Reporting Trials).

According to the calculation of the sample size, 110 patients were selected for the study. Ninety patients finally participated in the study and the CONSORT (Consolidated Standards of Reporting Trials, 2010) protocol was followed (Figure 1).

The criteria for inclusion was that patients had never worn an orthodontic treatment, patients were adolescent or young adults with permanent teeth, preferably between 14 and 45 years old, and the degree of crowding was measured using tooth size–arch length discrepancy (TSALD) and was negative, between −2.5 and −6.5, skeletal malocclusion class I and slight class II and III (ANB between 1 and 5 degrees) [14]. The criteria for exclusion was patients with periodontal disease, cavities or problems with pulpitis, significant psychiatric or physical issues, patients in need of orthodontic-surgery treatments, patients in a period of eruption and shedding, patients in need of exodontia and cases with severe discrepancies or average cases and patients under medication that influences pain at either a molecular or psychological level.

### 2.4. Procedure

The patients had the upper and lower brackets bonded in the first session. The tubes were also placed in the molars. The bonding was done directly with a conventional bonding technique for fixed multi-bracket appliances. The first arch used in all three groups was 0.014” (Nitinol-superelastic-3M-USA). In the GC and GS groups, the arch was ligated with metallic ligatures 0.010” (Cimbis Orthodontics, Madrid, Spain).

The patients were given different questionnaires for evaluating the pain and OHRQoL after putting the appliance in place. The first questionnaire evaluated the average pain using the VAS (Visual Analogue Scale) at different points in time: Four hours after starting the orthodontic treatment (T1), eight hours after (T2), twenty-four hours after (T3), two days after (T4), three days after (T5), four days after (T6), five days after (T7), six days after (T8) and seven days after (T9). The VAS is a simple straightforward method of measuring the intensity of pain experienced by the patients [15,16,17,18]. For the study, a 10 cm line, where 0 is the minimum pain and 10 is the maximum, was used on which the patients had to mark depending on the point in time where they were at that time. The second form was the Oral Health Impact Profile (OHIP-14) questionnaire that had to be filled out after the first month of treatment to analyse the level of impact on the oral health-related quality of life [19,20] to collect the frequency of appearance of problems/dysfunctions on a five-point Likert scale (0 = never, 1 = almost never, 2 = occasionally, 3 = relatively frequently, 4 = very frequently) [20,21]. That questionnaire was used, and its usage has been validated for the Spanish population by Montero et al. [20].

The patients started the study with good oral hygiene. The same hygiene guidelines were given to all three groups during the fixed orthodontic treatment. It was possible to be blind with the patients. Every patient had an assigned number so the operator who analysed the statistical data was blind, because they did not have personal information about the patients. In the three groups, the orthodontics were done by an expert operator with whom it was not possible to be blind, because the brackets have different shapes that can be observed visually. The randomisation of every patient in every group was done using an online programme to split them into three groups. (http://www.randomizer.org/form.htm, accessed on 4 January 2021).

The ANOVA test was used for the description of the variables analysed in the study. When the ANOVA test turned out to be statistically significant, the comparison between the groups was done using the post hoc Bonferroni test. Values of the *p*-value below 0.01 (*p* < 0.01) were considered to be highly significant results and *p*-values below 0.05 (*p* < 0.05) were considered significant. The SPSS version 20 (SPSS Inc. Chicago, Illinois) software was used for the statistical analysis of the data.

## 3. Results

The average age of the sample is 21.7 years old ± 7.5 years. The minimum age is 14 years old (in the three groups) and the maximum age is 44. There is a higher percentage of women than men in the three groups. In general, there were 56 women (62.2%) and 34 men (37.8%). The upper tooth size–arch length discrepancy has an average of −3.7 mm and the lower is −3.8 mm (Table 1).

### 3.1. Pain Analysis 

**Figure 2:**
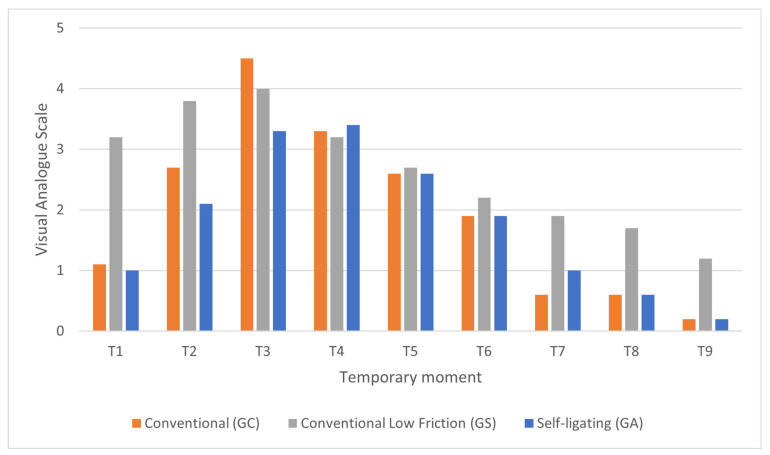
Analysis of pain at different times according to the Visual Analogue Scale (VAS) by groups.

After the analysis of the results of Table 2 using the ANOVA test, we found statistically significant results in pain levels according to VAS with a *p* < 0.01 in T1, T7 and T9 and with a *p* < 0.05 in T2 and T8, with the GS group being the one showing the highest pain values at all the time points (T1:3.2 ± 3, T2:3.8 ± 3.1, T7:1.9 ± 2.3, T8:1.7±2.4, T9:1.2 ± 2.3). The maximum peak of pain is reached at 24 hours in the GC group (4.5 ± 2) followed by the GS group (4.0 ± 2.9) and, at 48 hours, by the GA group (3.4 ± 1.7). Afterwards, pain progressively diminishes in the three groups until it reaches minimum pain values at the end of the week when treatment was started (Table 2, Figure 2).

### 3.2. Comparative Analysis of Quality of Life Related to Oral Health According to the Different Groups 

After the OHRQoL analysis, we observed how there is a greater negative impact in the GS group in the dimensions of physical pain (0.9 ± 0.8, *p* < 0.05), psychological discomfort (0.5 ± 0.7, *p* < 0.01), physical impairment (0.5 ± 0.7, *p* < 0.01) and total impact (1.4 ± 0.7, *p* < 0.01) (Table 3). The lowest OHRQoL value in the physical pain dimension (0.5 ± 0.6, *p* < 0.05) and the total impact (0.8 ± 2.2, *p* < 0.05) was for the GA group.

We did not find statistically significant differences in the functional limitation dimensions. It should be noted that the impact described by the patients in the dimensions of psychological impairment, social impairment and obstacles is null in all three groups in the study (Table 3).

## 4. Discussion

This study analysed the influence that friction has on design bracket type and pain perceptions in relation to oral health related to quality of life of the patients in the first month of fixed multi-bracket appliance treatment. Some earlier studies analyzed pain in orthodontics [22,23], and the majority of them compared conventional vestibular brackets and self-ligating brackets [24,25,26,27]. No studies were found that jointly analysed the pain experienced by patients and the OHRQoL between conventional brackets, conventional low-friction brackets and self-ligating brackets. There are, however, studies that analyse the quality of the impact of the orthodontics on patients’ quality of life [28,29,30]. Self-ligating brackets are considered to be low friction, but the difference with the Synergy brackets is that we do not need ligatures to join the arch with the bracket. In addition, the majority of the published studies that compare low-friction brackets are in vitro and analyse friction and force comparing conventional and self-ligating brackets, and they concluded that the low-friction brackets have less friction than the conventional ones, but also that not all of them are “low friction” [31]. Even though the manufacturers describe them, there are differences between the various low-friction bracket systems on the market. For that reason, the design of the low-friction bracket and the arch used need to be taken into account [32,33]. The majority of the in vitro studies found only compared self-ligating brackets with conventional brackets [34,35]. Therefore, the novelty of this study is to analyze the influence of the type of bracket and its design in terms of friction reduction, comparing two types of conventional and self-ligating brackets.

After analysing the published studies, we observed how the majority of the studies did not specify the slot used [36,37], and when the slot is specified, it is usually a 0.022” slot [38,39,40]. However, the slot is not taken into account when evaluating pain or quality of life. In our study, the decision was made to use the 0.022” slot in the three study groups so that variable would be homogeneous and bias would be avoided. We found other articles in the literature that analysed pain and quality of life with different kinds of appliances, however they used a 0.018′′ slot [41].

The majority of the papers analysed concluded that the pain experienced by patients reaches its maximum point between 1 and 2 days after starting treatment. The pain generally lasts two to three days and progressively diminishes until it reaches minimum levels a week after placing the fixed appliance [42,43,44,45]. We arrived at similar results with our analysis, where we observed peak pain at 24 hours in the conventional low-friction group (4.0 ± 2.9) and in conventional brackets (4.5 ± 2.0) and at 48 hours in the self-ligating group (3.4 ± 1.7). Both brackets have a low friction design, so the difference between the two in the relative friction generated must arise in the ligation and arch used. That increase may generate greater friction and, consequently, greater pain.

In contrast to other authors where statistically significant differences in pain experienced with conventional, low-friction and self-ligating brackets are not recorded [16,45,46], we did observe differences in the conventional low-friction (GS) group at four hours (3.2 ± 3, *p* < 0.01) and eight hours (3.8 ± 3.1, *p* < 0.05) in contrast to the self-ligating group with less pain (1.0 ± 0.8 y 2.1 ± 1.9 respectively) (Table 2).

In our study, we are going to analyse the OHRQoL a month after starting treatment. The majority of the studies conclude that discomfort and pain experienced by the patients in association with the treatment are going to negatively influence the oral health-related quality of life. Discomfort is greatest in the first stages, especially the first month of treatment; like pain, it diminishes in more advanced stages and even ends up improving until the end of treatment [47,48,49]. It would be necessary to carry out comparative studies with different bracket and aligner designs, as this is a factor to be taken into account in both orthodontic techniques.

The majority of patients believe that the orthodontic treatment has not had an influence on their daily tasks or on the feeling of having a less satisfactory life, nor has it incapacitated them to lead a normal life [50,51]. In other studies, it can be observed how the treatment improved quality of life during the period studied [52]. Nevertheless, there are studies which show OHRQoL worsens during the first stages of treatment [48,49]. In our study, we observed a greater negative impact in the GS group in the dimensions of physical pain (0.9 ± 0.8, *p* < 0.05), psychological discomfort (0.5 ± 0.7, *p* < 0.01), physical disability (0.5 ± 0.7, *p* < 0.01) and total impact (1.4 ± 0.7, *p* < 0.01). The lowest impacts in those dimensions are found in the self-ligating group of physical pain (0.5 ± 0.6, *p* < 0.05), psychological discomfort (0.5 ± 0.7, *p* < 0.01), physical disability (0.1 ± 0.3, *p* < 0.01) and total impact (0.8 ± 2.2, *p* < 0.01). Consequently, conventional brackets of the two groups (GC and GS) produced a more negative impact on quality of life due to the increase in friction caused by the tension of the ligature and greater periodontal pain alongside it.

As limitations to our work, we found that the distribution between gender was not equivalent between men and women (50% men/50% women); however, according to various authors, sex would not influence the amount of pain perceived in patients with multi-bracket orthodontic treatment [39,53]. Furthermore, some variables were not taken into account that could influence the study, like the patients’ surroundings, stress level, pain threshold or their previous experiences at the dentist’s office. In addition, the monitoring time was short and, insofar as the oral health-related quality of life, cultural differences and the social setting of the patient were not taken into account and also it was evaluated at a single point (the month the treatment started) [18]. Therefore, studies with long-term follow-up are necessary to confirm the short-term results shown in the present clinical study.

In our study, pain and the impact in OHRoL of the three orthodontic techniques were analysed in vivo and included in regards to other studies an analysis of the quality of life in reference to the friction of each type of bracket. In the future, we could consider including another type of low-friction and conventional orthodontic appliance for comparison and increase the number of participants, the monitoring time and be able to compare our data with other age ranges.

## 5. Conclusions

According to the results, we can conclude that there is a difference in terms of the pain perceived by patients and its influence on Oral Health-Related Quality of Life using different brackets with a different design influenced by friction. Conventional brackets cause more pain, despite having a low-friction design. Consequently, self-ligating brackets caused less pain and negative impact on quality of life in patients wearing fixed multi-bracket appliances.

## Figures and Tables

**Table 1:** Clinical and Sociodemographic Description by Treatment Group (*n* = 90).

			Brackets Conventional (GC) 0.022″ (*n* = 30)	Brackets Self-Ligating (GA) 0.022″ (*n* = 30)	Conventional Low-Friction (GS) 0.022″ (*n* = 30)
Age		Mean	20.4	22.7	22.1
		Sd.	5.9	7.2	9.2
Sex	Men	N	11	11	12
		%	36.7	36.7	40.0
	Women	N	19	19	18
		%	63.3	63.3	60.0
TSALD	Upper	Mean	−4.1	−4.0	−2.9
		SD	1.7	1.4	0.8
	Lower	Mean	−4.5	−3.9	−2.8
		SD	1.2	1.1	1.0

SD: Standard deviation. TSALD: Tooth size–arch length discrepancy.

**Table 2:** Comparison of Pain on the Visual Analogue Scale (VAS), between groups at Different Evaluation Times (*n* = 90).

Time	Conventional Brackets (GC) (*n* = 30)		Self-Ligation (GA) (*n* = 30)		Conventional Low-Friction (GS) (*n* = 30)	
	Average	SD	Average	SD	Average	SD
4 Hours (T1) **	1.1 ^A^	1.1	1.0 ^A^	0.9	3.2 ^B^	3.0
8 Hours (T2) *	2.7 ^a,b^	1.9	2.1 ^a^	1.9	3.8 ^b^	3.1
**24 Hours (T3)**	4.5	2.0	3.3	1.8	4.0	2.9
**2 days (T4)**	3.3	1.5	3.4	1.7	3.2	2.5
**3 days (T5)**	2.6	1.7	2.6	1.6	2.7	2.4
**4 days (T6)**	1.9	1.3	1.9	1.6	2.2	2.4
5 days (T7) **	0.6 ^A^	0.7	1.0 ^A,B^	1.0	1.9 ^B^	2.3
6 days (T8) *	0.6 ^a^	0.9	0.6 ^a^	0.9	1.7 ^b^	2.4
7 days (T9) **	0.2 ^A^	0.5	0.2 ^A^	0.6	1.2 ^B^	2.3

Statistically significant results: * (*p* < 0.05), ** (*p* < 0.01). Statistically significant differences between groups indicated by superscript letters (Bonferroni’s post hoc test). A = *p* < 0.01 vs. GC; a = *p* < 0.05 vs. GC. B = *p* < 0.01 vs. GS; b = *p* < 0.05 vs. GS.

**Table 3:** Comparison of the Impact on the Quality-of-Life Dimensions between Groups according to the OHIP-14 Questionnaire (*n* = 90). OHIP: Oral Health Impact Profile.

Dimensions	Conventional Brackets (GC) (*n* = 30)		Self-Ligating Brackets (GA) (*n* = 30)		Conventional Low-Friction Brackets (GS) (*n* = 30)	
	Average	SD	Average	SD	Average	SD
Functional limitation	0.1	0.3	0.2	0.4	0.3	0.5
	ANOVA F:1.26; fd:2; *p*-value: 0.29					
Physical Pain *	0.8 ^a,b^	0.7	0.5 ^a^	0.6	0.9 ^b^	0.8
	ANOVA F:3.80; fd:2; *p*-value: 0.03					
Psychological Discomfort **	0.1 ^A^	0.3	0.0 ^A^	0.2	0.5 ^B^	0.7
	ANOVA F:11.3; fd:2; *p*-value: 0.00					
Physical disability **	0.1 ^A^	0.3	0.1 ^A^	0.3	0.5 ^B^	0.7
	ANOVA F:8.09; fd:2; *p*-value: 0.00					
Psychological disability	0.0	0.0	0.0	0.0	0.0	0.0
Social Disability	0.0	0.0	0.0	0.0	0.0	0.0
Handicap	0.0	0.0	0.0	0.0	0.0	0.0
Total impact **	1.1 ^A^	0.8	0.8 ^A^	2.2	1.4 ^B^	0.7
	ANOVA F: 16.9; fd: 2; *p*-value: 0.00					

Statistically significant results: * (*p* < 0.05), ** (*p* < 0.01). Statistically significant differences between groups indicated by superscript letters (Bonferroni’s post hoc test). A = *p* < 0.01 vs. GC; a = *p* < 0.05 vs. GC. B = *p* < 0.01 vs. GS; b = *p* < 0.05 vs. GS.

## Data Availability

The data presented in this study are available on request from the corresponding author. The data are not publicly available due to privacy and ethical issues.
